# Nicotinamide Adenine Dinucleotide Phosphate Oxidases Are Everywhere in Brain Disease, but Not in Huntington’s Disease?

**DOI:** 10.3389/fnagi.2021.736734

**Published:** 2021-11-05

**Authors:** Luisana Villegas, Anne Nørremølle, Kristine Freude, Frederik Vilhardt

**Affiliations:** ^1^Department of Cellular and Molecular Medicine, University of Copenhagen, Copenhagen, Denmark; ^2^Department of Veterinary and Animal Sciences, Faculty of Health and Medical Sciences, University of Copenhagen, Frederiksberg, Denmark

**Keywords:** Huntington’s (disease), NADPH (nicotinamide adenine dinucleotide phosphate) oxidase, neuron, Huntingtin (HTT), NMDAR (NMDA receptor), LTP (long term potentiation), LTD (long term depression)

## Abstract

Huntington’s disease (HD) is an inherited neurodegenerative disorder characterized by neuronal loss and tissue atrophy mainly in the striatum and cortex. In the early stages of the disease, impairment of neuronal function, synaptic dysfunction and white matter loss precedes neuronal death itself. Relative to other neurodegenerative diseases such as Alzheimer’s and Parkinson’s disease and Amyotrophic Lateral Sclerosis, where the effects of either microglia or NADPH oxidases (NOXs) are recognized as important contributors to disease pathogenesis and progression, there is a pronounced lack of information in HD. This information void contrasts with evidence from human HD patients where blood monocytes and microglia are activated well before HD clinical symptoms (PET scans), and the clear signs of oxidative stress and inflammation in post mortem HD brain. Habitually, NOX activity and oxidative stress in the central nervous system (CNS) are equated with microglia, but research of the last two decades has carved out important roles for NOX enzyme function in neurons. Here, we will convey recent information about the function of NOX enzymes in neurons, and contemplate on putative roles of neuronal NOX in HD. We will focus on NOX-produced reactive oxygen species (ROS) as redox signaling molecules in/among neurons, and the specific roles of NOXs in important processes such as neurogenesis and lineage specification, neurite outgrowth and growth cone dynamics, and synaptic plasticity where NMDAR-dependent signaling, and long-term depression/potentiation are redox-regulated phenomena. HD animal models and induced pluripotent stem cell (iPSC) studies have made it clear that the very same physiological processes are also affected in HD, and we will speculate on possible roles for NOX in the pathogenesis and development of disease. Finally, we also take into account the limited information on microglia in HD and relate this to any contribution of NOX enzymes.

## Introduction

Microglia express NADPH oxidases (NOX) and in many neurodegenerative diseases (NDDs) microglia activation and the ensuing oxidant production through NOX2, together with proinflammatory response including cytokine secretion, play major roles in disease pathogenesis (Block et al., 2007; Tejera and Heneka, 2019). However, more recently NOX expression in neurons have attracted attention in physiological and pathological settings (Nayernia et al., 2014). Hence, changes in NOX activity in NDD’s are not necessarily restricted to microglia but may also take place in neurons as a cell-autonomous mechanism, all though evidence for direct involvement of neuronal NOX isoforms in NDD is still scarce.

Here, we will convey recent information about the function of NOXs in the Central Nervous System (CNS) and contemplate on the putative role of neuronal NOX in the neurodegenerative disorder Huntington’s Disease (HD). We will focus on NOX-produced reactive oxygen species (ROS) as a signaling molecule in/among neurons and the specific roles of NOXs in important processes in the synapses and neurites. We will thus compare the processes where NOXs are involved to the pathogenic mechanisms of HD, asking if NOXs are likely to be players in the initial synaptic dysfunction that characterizes HD, as well as later in disease progression. Given the high expression of NOX in microglia, we will also review the limited literature of NOX derived ROS in this particular cell type in relation to HD.

## NOX and Oxidants: Oxidative Stress Versus Redox Signaling

Before we delve into the intricacies of NOX activity and oxidant production in neurons it is justified to briefly put into perspective the phenomenon of oxidative eustress versus oxidative stress. Oxidative stress is a condition with an imbalance in oxidant and antioxidant production, such that antioxidant defense mechanisms are overwhelmed by excessive oxidants. As such, oxidative stress is thought to be an inherent property of many neurodegenerative diseases (Singh et al., 2019). In the early days, oxidative stress was synonymous with the indiscriminate and irreversible oxidation and destruction of biomolecules and detected as molecular signs of oxidative modification of biomolecules (nitrosylation, sulfonation, carbonylation, and peroxidation). Today we know that under physiological circumstances cellular redox balance (oxidative eustress) is tightly maintained to allow oxidants to act as signaling molecules typically by their reversible oxidation of low pKa cysteines in target redox proteins, with a wide spectrum of functions that are still not fully discovered (Sies and Jones, 2020). Therefore, the concept of oxidative stress has since been expanded to account for the fact that even small perturbations of local or global redox milieu can seriously disrupt redox signaling circuits (Sies, 2014).

The CNS itself is highly susceptible to oxidative stress due to its fast rate of oxygen consumption and high iron content (Bresgen and Eckl, 2015), resulting in an increased generation of ROS. In addition, the antioxidant machinery that exists to counteract oxidative stress has lower levels of expression in the CNS, and in neurons particularly (Kamat et al., 2008; Teleanu et al., 2019). This relatively low neuronal antioxidant level could be due to a tradeoff; as ROS is required for exit from the neurogenic stem cell niche and neuronal induction (Nayernia et al., 2017), nerve cells habitually express low levels of nuclear factor erythroid 2-related factor 2 (NRF2) (Bell et al., 2015), a major transcriptional regulator of antioxidant genes (Hardingham and Do, 2016).

## Huntington’s Disease

HD is an autosomal dominant inherited, neurodegenerative disease caused by an expansion of a CAG repeat in exon 1 of the HD gene, encoding an elongated poly-glutamine stretch in the huntingtin protein (The Huntington Disease Collaborative Research Group, 1993). Symptoms of HD include motor, psychiatric and cognitive changes usually emerging in midlife (with an earlier onset in those patients with larger poly-glutamine expansions), progressing to eventually cause fatal multi-system failure. Death of neurons, in particular striatal medium spiny neurons (MSNs) and cortical projection neurons but also neuron populations in other brain regions, is a prominent feature in late-stage HD. The MSNs are gamma-aminobutyric acid-ergic (GABAergic) inhibitory neurons receiving glutamatergic input from cortex, as well as dopaminergic input from the substantia nigra pars compacta (Blumenstock and Dudanova, 2020). MSNs affect the activity of neurons in the thalamus and cortex, thereby regulating the initiation and execution of movements. MSNs are divided in two classes, where the MSNs of the direct pathway project to output nuclei in the substantia nigra pars reticulate and the internal part of the globus pallidus (which connect to the thalamus), whereas the MSNs of the indirect pathway project to inhibitory neurons in the external part of the globus pallidus. These inhibitory neurons in turn affects the same output nuclei as the direct pathway, leading to opposing effect on the activity of these: the MSN direct pathway inhibits, and the MSN indirect pathway activates, the GABAergic neurons of the substantia nigra pars reticulate and the internal part of the globus pallidus (Blumenstock and Dudanova, 2020). In HD progression, the MSNs of the indirect pathway are the first to degenerate, which correlate well with the prominent involuntary movements (hyperkinesia) which are among the early symptoms of HD.

Before reaching final stage of neurodegeneration, at which symptoms of HD are evident and widespread, patients endure an extended period of gradual disease progression. Increasing neuronal dysfunction, specifically synaptic dysfunctions such as impaired synaptic plasticity and dysregulation of synaptic transmission are observed, most likely starting already at the prodromal, pre-symptomatic phase (Smith-Dijak et al., 2019). In particular, aberration of cortico-striatal connections are prominent and may (at least in part) explain the early hyperkinetic motor symptoms of HD (Plotkin and Surmeier, 2015). Because motor symptoms of the disease manifest before there is overt nerve cell death, HD is sometimes referred to as a ‘synaptopathy’. Indeed, both cortical and striatal neurons of HD mouse models display synaptic dysfunction at very early, pre-symptomatic stages (reviewed in Cepeda and Levine (2020)). These functional changes are supported by proteomic analysis, in which early HD-related changes predominantly occur in proteins involved in synaptic function (Skotte et al., 2018; Sapp et al., 2020). According to the “dying-back” theory, such early synaptic changes can lead to synapse and neurite degeneration and loss, which may eventually result in neuronal death (Han et al., 2010).

Huntingtin (Htt) is ubiquitously expressed and localizes mainly in the cytoplasm, but also in the nucleus; within the cell, huntingtin associates with numerous cellular structures and molecules (Saudou and Humbert, 2016). Htt has a tremendous number of protein interaction partners (> 100), which perhaps point to a general function of Htt as scaffolding protein, and in part dictates the many proposed roles of Htt as well as the subcellular localization of Htt. In neurons Htt can be found along neurites and in terminals, in part due to its interaction with the dynein-dynactin complex (Rui et al., 2015), which drives vesicular transport along microtubules. Htt has a major role in the regulation of autophagosome (Wong and Holzbaur, 2014; Rui et al., 2015), endosomal (Caviston et al., 2007), or stress granule (Ma et al., 2011) dynamics and positioning, and the very same processes and organelles (among others) are affected adversely by mHtt (Gunawardena et al., 2003; Morfini et al., 2009; Martinez-Vicente et al., 2010; Wong and Holzbaur, 2014). Htt also interacts with the actin cytoskeleton through binding to a-actinin, an actin anchoring and cross-linking protein, and in fibroblasts co-localizes with actin stress fibers and adhesion structures (Tousley et al., 2019a). In mHtt-expressing cells, a large fractional area of the cytosol is occupied by spherical inclusion bodies of aggregated mHtt surrounded by markers of autophagy (p62/SQSTM1) and intermediate filaments. Inclusion formation can have negative effects on organelles (Bauerlein et al., 2017), trafficking systems (Woerner et al., 2016), and cause the sequestration of other glutamine-rich and prion domain-containing proteins (Ramdzan et al., 2017) but may also have cytoprotective function (Miller et al., 2010).

In HD patients, mutant huntingtin is expressed throughout life, including during embryogenesis (Saudou and Humbert, 2016). In line with this, the expression of mutant huntingtin has been shown to affect not only adult neuronal function, leading to neurodegeneration, but also neurodevelopment including neuronal differentiation and establishment of connectivity through neurite outgrowth (Conforti et al., 2018; Barnat et al., 2020; van der Plas et al., 2020). Intriguingly, mouse models expressing mutant huntingtin solely during embryonic and early development develop HD-like symptoms (Molero et al., 2016). As hypothesized by Lu and Lu, these data indicate that neurodevelopmental dysregulation may play an important role in HD pathogenesis developing in adulthood (Lu and Lu, 2021).

Although the initial cause of the pathology is known – expression of huntingtin carrying an expanded poly-glutamine stretch – the downstream pathogenic processes are still not well understood. As excellently reviewed by Bates et al., mutant huntingtin has been shown to lead to (among others) transcriptional dysregulation, impairment of axonal vesicular transport, synaptic dysfunction and impaired mitochondrial function (Bates et al., 2015). In both human post mortem HD brain (Sorolla et al., 2008) and HD animal models (Bogdanov et al., 2001) there are tell-tale signs of oxidative stress, but focus has almost exclusively been on mitochondria-generated ROS (Zheng et al., 2018; Fão and Rego, 2021) ignoring the major oxidant producing enzymes, the family of NOXs (Brown and Borutaite, 2012; Lambeth and Neish, 2014; Sies and Jones, 2020).

## The Family of Nicotinamide Adenine Dinucleotide Phosphate Oxidases

NOXs are transmembrane, superoxide-producing enzyme complexes. Once activated, the NOX complexes transfer electrons from nicotinamide adenine dinucleotide phosphate (NADPH) in the cytosol to molecular oxygen on the other side of the membrane, thus producing superoxide (O_2_. ^–^) in the extracellular space or the topologically identical luminal space of organelles and vesicles. Superoxide quickly dismutation to hydrogen peroxide spontaneously, or more commonly at high levels of oxidants, via superoxide dismutase activity.

**FIGURE 1:**
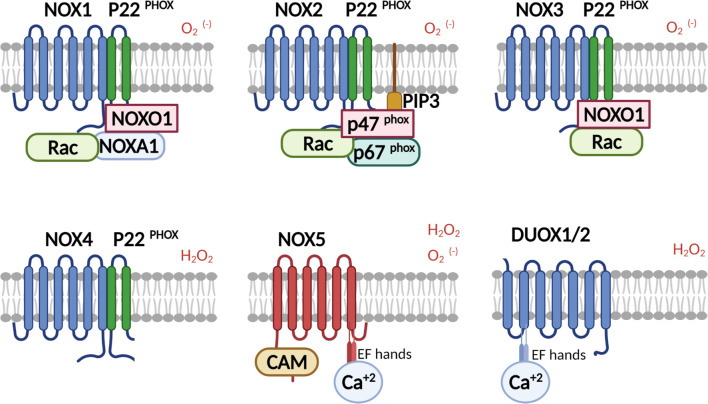
Schematic representation of the family of NADPH oxidases. NOX isoforms and regulatory subunits: NOX1-3 are activated in the presence of cytosolic phox proteins, where PIP3 serves as a membrane anchor for p47^phox^ in the case of NOX2 activation. NOX1-3, and NOX5 release superoxide (in some cases NOX5 also hydrogen peroxide), whereas NOX4 and the DUOXes release only hydrogen peroxide. NOX4 is constitutively active and is regulated by expression. NOX5 and DUOX1/2 are calcium dependent through calcium-calmodulin binding or by the direct binding of Ca^2+^ to EF-hands in DUOXes, respectively.

The NOX family is comprised of 7 isoforms (NOX1-5 and DUOX1-2) (Figure 1; Bedard and Krause, 2007). The first prototypical member of the family to be characterized was the classical phagocyte NADPH oxidase, now called NOX2, consisting of a membrane-anchored flavocytochrome b_558_, itself composed of two membrane proteins gp91^phox^ (also confusingly referred to as NOX2), containing heme redox relay stations for electron transfer, and p22^phox^. Activation of the NOX2 enzyme involves further recruitment of four cytosolic proteins which translocate to the membrane: p67^phox^ and the small GTP-binding protein Rac1/2, which together regulate electron transfer from NADPH to redox centers in gp91^phox^, and p40^phox^ or p47^phox^ as organizers of assembly (Vignais, 2002). While introduction of dominant active Rac1 is sufficient to induce NOX activation in a heterologous cell model with NOX2 overexpression (Price et al., 2002), it is insufficient in phagocytes, where the critical step in NOX2 activation (at least in microglia) is phosphorylation of p47^phox^ (Roepstorff et al., 2008). Multiple phosphorylation events on serines uncovers the latent SH3 domains of p47^phox^, making them available for binding to p22^phox^ (Sumimoto et al., 1996). In addition, PX domains contained in the SH3 domains of p47^phox^ and p40^phox^ contributes to membrane recruitment through binding to phosphoinositide lipids (Zhan et al., 2002; Ago et al., 2003).

NOX1 consists of the membrane-anchored subunits NOX1 and p22^phox^, which interacts with NOXO1 and NOXA1, homologs of p47^phox^ and p67^phox^, respectively, as well as Rac. Likewise, NOX3 interacts with p22^phox^ in addition to NOXO1 (Cheng et al., 2004). In contrast, the NOX4-p22^phox^ complex, as well as the NOX5, DUOX1 and DUOX2 isoforms can be activated independently of cytosolic phox proteins (Bedard and Krause, 2007). NOX4 is thought to be constitutively active, while NOX5 and DUOX1/2 have N-terminal extensions containing 2 to 4 EF-hand Ca^+2^ binding domains allowing their activation through calcium sensing (Banfi et al., 2004) (see Figure 1 for schematic representation of NOX isoforms). Both NOX4 and the DUOX enzymes release hydrogen peroxide rather than superoxide.

NOX2, the classical phagocyte NADPH oxidase, is well known for its bactericidal role in innate immune defense (Nauseef, 2019), but with the advent of the NOX superfamily it was quickly realized that these enzymes occupies an important role as oxidant producers in an intricate network of cellular redox signaling circuits. Hydrogen peroxide is here believed to be the redox-relevant signaling messenger, and controls the activity of target proteins typically by the reversible oxidation of low pKa cysteines or metal centers (Sies and Jones, 2020; Petersen et al., 2021). The redox targets are diverse, but for the purpose of this review it is worth mentioning that many ion channels, kinases, and phosphatases are regulated or modulated by hydrogen peroxide (Winterbourn, 2013; Sies, 2014; Sies and Jones, 2020).

In the CNS, mainly NOX2 and NOX4 isoforms are expressed under basal conditions, while other NOX isoforms can be induced by stimulation (Massaad and Klann, 2011; Nayernia et al., 2014). Different neuronal populations (cortical, hippocampal, hypothalamic paraventricular nucleus neurons, cerebellar granule neurons, neurons of the sympathetic system, parvalbumin-positive GABAergic interneurons) mainly express the phagocyte NOX2, but dopaminergic neurons express NOX1, and dorsal root ganglion neurons (DRGs) express NOX4 (Massaad and Klann, 2011; Nayernia et al., 2014; Sorce et al., 2017). Glial cells and the vascular compartment also express NOX isoforms (Nayernia et al., 2014), and in particular microglia express NOX2. Because only NOX2 function in CNS has been addressed with some weight, we will in the following sections focus on neuronal expression of this isoform in CNS neurons and microglia.

## Subcellular Localization of NOX in Neurons

Virtually all cells of our body express NOX isoforms. Many cell types express more than one NOX isoform, and despite their high homology, they localize differentially in a cell type-specific manner. For example, in endothelial cells, NOX1 is present in caveolae while NOX4 segregates to focal adhesions (Helmcke et al., 2009); in microglia, NOX1 seems to reside in lysosomes (Cheret et al., 2008), while NOX2 is localized to agonist-responsive secretory vesicles and the plasma membrane (Ejlerskov et al., 2012). Because of the large and polarized shape of neurons it is reasonable to assume that the same can occur in neurons, such that separate redox signaling circuits can function simultaneously in the same cell (see discussion in Petersen et al. (2021)). No sorting receptors for NOX have been identified, and there are very few clues to the differential and cell-specific subcellular distribution of the different NOX isoforms, as very few interaction partners of NOX family members have been identified (Park et al., 2004; Ikeda et al., 2005; Gianni et al., 2009). In microglia, the small GTPases Rab27A/B determine the distribution of intracellular to cell surface-resident NOX2 (Ejlerskov et al., 2012), which in essence determines the release to the surroundings of either hydrogen peroxide or superoxide, respectively, because of their different membrane permeabilities.

In the following sections, we have compiled the available data of NOX2 localization in neurons (summarized in Table 1). The data have been compiled from ultrastructural studies, most of them performed by the groups of Iadecola and Wilkens. The immunolocalization is of high quality, and in several cases quantitation of NOX2 subunits distribution was performed (Glass et al., 2006; Coleman et al., 2013). The expression and subcellular distribution of other NOX family members in neurons has not been analyzed in detail.

**TABLE 1:** The subcellular distribution of NOX2, p22^phox^, and p47^phox^ observed by immunolabeling in neurons (Wang G. et al., 2004; Glass et al., 2006; Brennan et al., 2009; Girouard et al., 2009; Minnella et al., 2018).

Somata	Plasma membrane	gp91^phox^ (Wang G. et al., 2004; Glass et al., 2006), p47^phox^ (Coleman et al., 2013)
	Mitochondria	gp91^phox^ (Wang G. et al., 2004; Girouard et al., 2009; Coleman et al., 2013)
	MVBs	gp91^phox^ (Glass et al., 2006), p47^phox^ (Coleman et al., 2013)
	Vesicular organelles of the cytoplasm	gp91^phox^ (Glass et al., 2006; Girouard et al., 2009), p22^phox^, p47^phox^ (Glass et al., 2006) p47^phox^ (Coleman et al., 2013)
	Rough ER	gp91^phox^ (Glass et al., 2006), p47^phox^ (Coleman et al., 2013)
	Golgi Complex	gp91^phox^ (Wang G. et al., 2004), p22^phox^ (Glass et al., 2006), p47^phox^ (Glass et al., 2006)
	Cytosol	p47^phox^ (Coleman et al., 2013)

Dendrites	Plasma membrane	gp91^phox^ (Glass et al., 2006), p47^phox^ (Coleman et al., 2013)
	Perisynaptic plasma membrane	gp91^phox^ (Glass et al., 2006; Wang et al., 2015), p22^phox^, p47^phox^ (Coleman et al., 2013) (Glass et al., 2006; Coleman et al., 2013)
	Mitochondria	gp91^phox^ (Glass et al., 2006), p22^phox^ (Glass et al., 2006), p47^phox^ (Coleman et al., 2013)
	Smooth ER	gp91^phox^ (Wang G. et al., 2004)
	MVBs	gp91^phox^ (Wang G. et al., 2004)
	Vesicular organelles of the cytoplasm	gp91^phox^ (Glass et al., 2006), p22^phox^, p47^phox^ (Glass et al., 2006)
	Undefined endomembrane beneath synapse	gp91^phox^ (Glass et al., 2006; Girouard et al., 2009), p47^phox^ (Coleman et al., 2013)
	Cytosol	gp91^phox^ (Glass et al., 2006; Coleman et al., 2013)

Axons	The plasma membrane of an axon terminal	gp91^phox^ (Wang et al., 2015)
	Clear vesicles of an axon terminal	gp91^phox^ (Wang G. et al., 2004; Wang et al., 2015)

*The most thorough studies used immunogold labeling and immunoperoxidase staining to reveal co-localization of cytb_558_ and p47^phox^ in mainly intracellular vesicular organelles, or cell surface in dendrites of the nucleus tractus solitarius (Glass et al., 2006); or localization of p47^phox^ in the paraventricular thalamic nucleus (Coleman et al., 2013).*

### NOX2 in the Soma of Neurons

NOX2 can be found at various sites in neuronal cell bodies including the plasmalemma, rough ER, smooth ER, Golgi Complex, mitochondria, multivesicular bodies (MVBs) and vesicles (Wang G. et al., 2004; Girouard et al., 2009; Coleman et al., 2013). However, detection of gp91^phox^ and p22^phox^ at some of these sites is likely due to their biosynthesis.

### NOX2 in Neurites

In developing neurons NOX2 localizes to growth cones, including filopodia and lamella (Munnamalai et al., 2014; Terzi et al., 2021), as we will later discuss. Here, we will mainly focus on mature neurons *in vivo*, where most ultrastructural studies have been performed. Within dendrites, p22^phox^, p47^phox^, and gp91^phox^ are predominantly found on intracellular vesicular organelles, but also at the plasma membrane close to dendritic spines (Wang G. et al., 2004; Glass et al., 2006; Girouard et al., 2009). In a quantitative study, the majority of NOX2 labeling (gp91^phox^, p22^phox^, and p47^phox^ labeling) was in fact associated with dendrites and less with soma, axons, and terminals (Glass et al., 2006). Intracellularly, p47^phox^ and gp91^phox^ can be detected in association with vesicular organelles and mitochondria (Glass et al., 2006). Additionally, the gp91^phox^ subunit has been found on membranes resembling smooth endoplasmic reticulum (Glass et al., 2006) and in multivesicular bodies (endosomes) (Wang G. et al., 2004).

### NOX2 in Synapses

Two separate studies have used electron microscopy to dissect the subcellular distribution of NOX2 in neurons of the nucleus tractus solitarius (brain stem). One study shows that immunogold-labeled NOX2 is present in pre-synaptic axon terminals, possibly belonging to the vagus nerve. More specifically, NOX2 is found at the plasma membrane and associated with small clear vesicles of the terminal and some MVB’s (Wang G. et al., 2004). A different study that systematically quantified the p47^phox^, gp91^phox^, and p22^phox^ distribution concludes that only a fraction of these subunits localize to axon terminals or axons in general (Glass et al., 2006). Both studies, however, agree that gp91^phox^, p22^phox^, and p47^phox^ can be found on or just beneath the peri-synaptic portions of the post-synaptic (dendritic) plasma membrane (Wang G. et al., 2004; Glass et al., 2006). Interestingly, in a quantitative approach, angiotensin II stimulation was shown to induce p47^phox^ translocation from cytosol to undefined endomembranes in close proximity to post-synaptic membranes of non-vassopressin hypothalamic paraventricular neurons (Coleman et al., 2013). Taken together, NOX2 can be found at synaptic sites, especially in the post-synaptic (dendritic) part, which is also confirmed by biochemical analysis of synaptosome preparations (isolated pre- and post-synaptic structures) prepared from hippocampal neurons (Tejada-Simon et al., 2005; Brennan et al., 2009), as well as synaptosome preparations from aged mice (Ali et al., 2011).

## NOX and Mutant Huntingtin; A Direct Interaction?

While there is good evidence for expression of NOX2 in cortical projection neurons (Massaad and Klann, 2011; Nayernia et al., 2014), some of which project to innervate striatal GABAergic MSNs, there are no data on the expression of NOX isoforms in the MSNs themselves. One of the few studies that relates expression of the HD mutation and cellular pathology to NOX expression applied immunoprecipitation which suggests a direct interaction between NOX2 and mutant huntingtin (Bertoni et al., 2011). This study by Bertoni et al. indicated that mutant huntingtin from patient fibroblasts was selectively sequestered in cholesterol- and glycosphingolipid-enriched membrane microdomains (lipid rafts) (Bertoni et al., 2011), which are central platforms for organization and signaling. Interestingly, NOX2 also segregates to lipid rafts (Vilhardt and van Deurs, 2004). Moreover, using an inducible polyQ expression system on PC12 cells, they propose that expanded polyQ proteins interact with gp91^phox^, as indicated by co-immunoprecipitation, which promotes NOX2-related oxidative stress in the cells (Bertoni et al., 2011). In accordance with the above, expanded polyQ tracts in Ataxin-7 have been shown to activate NOX in a cell model (Ajayi et al., 2012). Remarkably, treatment of polyQ-GFP-expressing PC12 cells with gp91ds-Tat (inhibiting NOX2 complex assembly) not only prevented the formation of new polyQ aggregates (which are typical of polyQ expanded diseases such as HD), but also dissolved the already existing ones (Bertoni et al., 2011). Moreover, a single *in vivo* study shows that instrastriatal injection of quinolinic acid in Wistar rats causes HD like symptoms, as well as increased NOX activity. Inhibition with apocynin resulted in less ROS production and prevented excitotoxicity (Maldonado et al., 2010).

These few studies constitute the entirety of NOX-related research on a HD background, which compared to the wealth of information available on the role of NOX-derived oxidants (mainly from microglia) in other NDDs is surprisingly limited. In the following sections of this review, we will speculate on the possible pathways in HD pathogenesis where NOX could play a role. Therefore, we will look into the known physiological functions of NOXs in neuronal development, differentiation, and synaptic function and plasticity with the purpose of discussing and speculating where in these processes NOXs may be contributing to the pathogenesis of HD. For a wider view of NOX roles in the CNS, we refer to a recent review by Terzi and Suter (2020).

Before we venture into the literature addressing any role of NOX isoforms in neuron function *in vivo* or *in vitro*, a technical *caveat* is warranted. In many studies, various synthetic inhibitors purported to specifically inhibit NOX2 have been used, and the results should be interpreted with caution. For example, apocynin, purported to inhibit p47^phox^ translocation to the membrane, is most likely a general antioxidant and has no specificity for NOX2 (Sorce et al., 2017). Therefore, the use of particularly knockout animal models of NOX isoforms, but also shRNA knockdown or expression of dominant negative constructs of NOX subunits, is the golden standard to affirm specific involvement of any NOX isoform in a given process. The use of gp91 ds-tat is also generally accepted by most as being a specific inhibitor of NOX2. The inhibitor consists of a 9 amino acid peptide of gp91^phox^ (amino acids 86–94) which binds to p47^phox^, therefore preventing endogenous complex assembly. The tat portion is a 9 amino acid sequence of the HIV-tat transport region, allowing it to enter into the cells (Rey et al., 2001).

## Role of NOXs in Neurogenesis/Neurodevelopment

A few studies have delineated a role for NOX2 in neurogenesis in the subventricular zone (SVZ) of the mammal brain, and in human iPSC-derived neurons. Thus maintenance of the neurogenic niche and neuroprogenitor proliferation depends on a sustained oxidant production through NOX2, which is expressed by neurogenic stem cells and their progenitors (Yoneyama et al., 2010; Le Belle et al., 2011; Kokovay et al., 2012; Nayernia et al., 2017). Some details have been unraveled. Thus, vascular cell adhesion protein 1 (VCAM1) expression in neuronal stem cells is essential to uphold the neurogenic niche, and VCAM1 in turn activates NOX2 to produce superoxide (Kokovay et al., 2012). Others have identified upregulation of Akt signaling as important for neuroprogenitor proliferation. NOX2-derived oxidants inhibit the opposing phosphatase and tensin homolog (PTEN) (although only chemical NOX2 inhibitors were used to arrive at this conclusion) (Le Belle et al., 2011), and Akt membrane association and activation is itself redox-modulated (Su et al., 2019). The most careful study has been performed using knock-out models to substantiate NOX involvement both *in vivo* and *in vitro* during iPSC differentiation to neurons (Nayernia et al., 2017). The authors find that gp91^phox^, specifically, is strongly upregulated to allow the passage from pluripotency to neural induction and then disappears during neuronal differentiation and maturation. Interestingly, the downregulation of p22^phox^ is less impressive which could hint at a role for other NOX family members in differentiated cells.

## Regulation of the Neurogenic Stem Cell Niche in HD

While HD is a neurodegenerative disease, it is also apparent that there is a neurodevelopmental aspect to it, which is played out in iPSC and embryonic stem cell (ESC) models of disease and in animal models. As mentioned earlier, a study using a mouse model conditionally expressing mutant huntingtin (97Q) only until postnatal day 21 showed that many of the pathological phenotypes observed in other HD mouse models are recapitulated in the aged mice, indicating that mutant huntingtin expression during development is sufficient for disease induction (Molero et al., 2016). It has been shown that mutant huntingtin expression is associated with a lower rate of progenitor cell division in isogenic ESC models (Ooi et al., 2019), as well as in cortical tissue from HD carrier fetuses at gestation week 13 (Barnat et al., 2020). Moreover, HD neural progenitor cells (NPCs) enter earlier into neuronal lineage fate, in comparison to their WT controls. Studies using mutant huntingtin knock-in cell lines showed that NPCs displayed impaired lineage restriction, lower proliferation rates, and aberrant MSN subtype specification (Molero et al., 2016). In unrelated studies by Xu et al. (2017), they corrected the huntingtin mutation in isogenic lines (Control: CAG33, mutant: CAG180) with a CRISPR-Cas9 and piggyBac transposon-based approach. Correction of the mutation reverted the phenotype to proper establishment of neural rosettes in culture, as opposed to what was seen in the non-modified CAG180 line. The latter was a specific effect of the HD mutation given the same genomic background of the isogenic lines (Xu et al., 2017). Given the role of NOX2 in progression from the stem cell niche to neuronal induction (Nayernia et al., 2017), it is possible that derangement of NOX activity could contribute to these phenomena of altered neuronal induction and differentiation in HD.

## Role of NOXs in Neurite Growth, Specification, and Connectivity During Development and After Nerve Injury

A few *in vitro* studies first indicated a role for NOX activity in neurite outgrowth and extension in the commonly used catecholaminergic PC12 nerve cell line (Suzukawa et al., 2000; Ibi et al., 2006). But only recently has the involvement of NOX in neurite extension, specification (dendrite or axon), and targeting been addressed in different animal models or *in vitro* models of nerve cell sprouting (Munnamalai et al., 2014; Olguin-Albuerne and Moran, 2015; Wilson et al., 2015, 2016; Weaver et al., 2018; Terzi and Suter, 2020).

One of the first studies supporting a role of NOX in neurite growth demonstrated the presence of gp91^phox^ in growth cones of *Aplysia* bag cell neurons. NOX2 was shown to orchestrate actin dynamics in growth cones, since chemical NOX2 inhibition reduced F-actin levels, and lowered retrograde transport, accompanied by shorter neurites (Munnamalai et al., 2014). When neurite growth was induced, an increase in co-localization of p40^phox^ with gp91^phox^ at apCAM (neural cell adhesion molecule (NCAM) homolog) adhesion sites was observed, indicating NOX2 complex formation and activation.

Related studies showed that chemical or genetic inhibition of NOX2 activity significantly diminished neurite outgrowth in primary cerebellar granule neurons (Olguin-Albuerne and Moran, 2015). *In vitro* peaks of ROS production correlated with high expression and activity of NOX1 and NOX2; also, the majority of hydrogen peroxide production, as measured by the hydrogen peroxide-specific biosensor Hyper, was in focal sites of axonal and dendritic growth cones, as well as in filopodia, with the highest peak before elongation of the latter.

In rat primary hippocampal neurons, NOX2 inhibition by chemical, biological, or genetic (dominant negative -p22^phox^ expression or p47^phox(–/–)^ mice) means, resulted in decreased axonal growth and loss of cell polarization *in vitro* (Wilson et al., 2015). Expression of dominant negative -p22^phox^ (which would inhibit NOX1-3) also interfered with the lamellae area (region of outgrowth of axons and minor neurites). NOX2 inhibition also reduced the number, length and lifetime of filopodia on axonal tips.

Further studies from the same group using the fluorescent Hyper biosensor (specific for hydrogen peroxide, and amenable for imaging) revealed that the highest hydrogen peroxide production was found at the periphery of the soma as well as at the axonal tip (Wilson et al., 2016). This study also represents the only documented example of a gain-of-function effect (increased neurite growth) when NOX2 activity is increased, in this case by p47^phox^ over-expression. NOX2 is also present in the growth cones of retinal ganglion cells of zebra fish and is required for proper axonal targeting and connection with the optical tectum (Weaver et al., 2018).

Altogether, the studies suggest a positive role for physiological levels of oxidant production by NOX2 in neuritic outgrowth and arborization in diverse neuronal populations, which may also translate to other neuronal subtypes of the CNS. Interestingly, PC12 cells express both NOX1 and NOX2, which are differentially regulated during NGF-induced differentiation such that NOX2 promotes neurite extension, and is down-regulated during the process, while NOX1 opposes neurite extension and is upregulated (Ibi et al., 2006), suggesting different signaling roles depending on the specific NOX isoform. However, the CNS of chronic granulomatous disease (CGD) patients, who lack NOX2 activity, and NOX2 knock-out animal models, develop with only mild cognitive deficits (Kishida et al., 2006). Thus, while a certain redundancy between NOX isoforms can perhaps be expected (Weaver et al., 2018), it seems prudent to conclude that NOX2-mediated redox signaling is a modulating factor, but physiologically important (Terzi et al., 2021), among many, in the development of neurite outgrowth, polarity, targeting, and connections in the brain.

NOX may also have neuroprotective roles. NOX2 activity is required in at least one example of axonal regeneration following peripheral nerve injury. Studies by Hervera and colleagues on mechanically lesioned dorsal root ganglia (DRGs) neurons from mouse, showed that oxidants (hydrogen peroxide) are required for axonal regrowth after nerve injury, indicating a neuroprotective role (Hervera et al., 2018). Unexpectedly, the NOX2 complex is ‘delivered’ to the DRGs by tissue macrophages by emission of CD63-positive exosomes that contain the full package of NOX2 subunits and are oxidant production proficient. The exosomes are internalized by the DRGs, and traffic to Rab7- and Trk-positive signaling endosomes. Interestingly, only those exosomes deriving from WT bone-marrow-derived macrophages (BMDMs), but not Ncf1−/− (human homolog of p47^phox^) or Nox2−/− BMDMs, were capable of inducing axonal regrowth after nerve injury (Hervera et al., 2018).

## Axonal Pathfinding in HD Neurodevelopment

In neurodevelopment axonal pathfinding is crucial to create a proper connectivity in the brain, and here, as is described above, NOX2 has been shown to have a role in growth and neurite guidance of developing neurons, and to localize in growth cones, associated with site specific peaks of ROS production (Olguin-Albuerne and Moran, 2015; Weaver et al., 2018; Terzi et al., 2021). Recent studies have shown that NOX acts as downstream effector of guidance cue molecules in developing neurons, with NOX2 mutants displaying aberrant axonal projections (Terzi et al., 2021). Interestingly, other oxidoreductases may be involved in pathfinding. Members of the cytosolic MICAL (molecule interacting with CaL) family of oxidoreductases have been shown to exert an essential function in mediating semaphorin–plexin repulsive axon guidance and cell morphological changes by direct redox modification (cysteine oxidation by hydrogen peroxide) of actin (Hung et al., 2010, 2011). The modification greatly increases affinity of actin for cofilin, an actin-severing protein, and promotes growth cone collapse.

RNAseq analysis of HD iPSCs-derived GABAergic neuronal cultures show a decreased expression of genes that are key for correct axonal guidance (HD IPSCs consortium). Moreover, Htt is required for newborn neuron migration and for the multipolar to bipolar transition during corticogenesis, as three-dimensional reconstructions of dendritic trees of conditional knockout Htt cells (timely done when developing neurons have migrated to the different cortical layers targets, and synaptic connectivity needs to be established) resulted in a decrease of the dendritic length and dendritic branching in comparison to control cells (Barnat et al., 2020). Furthermore, in HD human iPSCs derived cortical neurons, longer CAG repeats correlated with shorter neurites. The latter was confirmed by alterations in transcriptomics that corresponded to altered cellular morphology (Mehta et al., 2018). Therefore, it would be interesting to assess if mutant huntingtin changes the pattern of expression and/or activity of NADPH oxidases during neurodevelopment.

## NMDAR Signaling and NOX2 Activation

**FIGURE 2:**
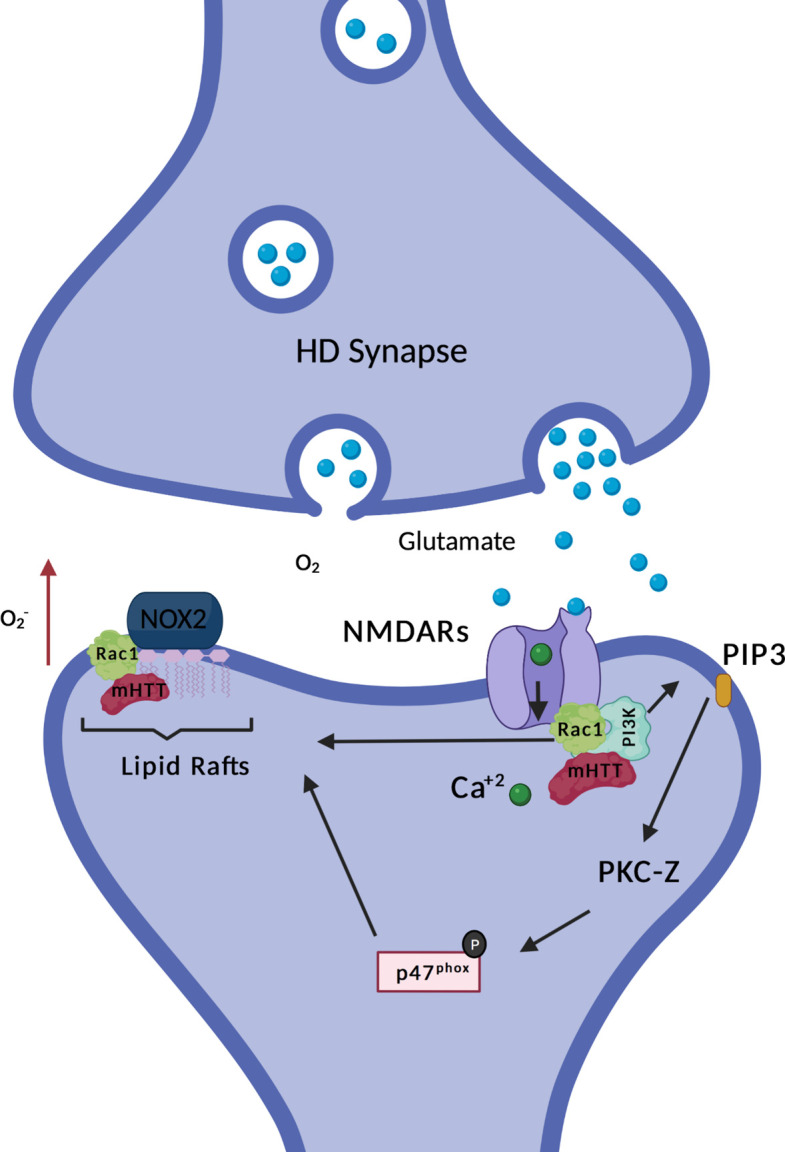
HD and NOX2 interaction. Non-ionotropic activation of PI3K (explained with more details in Figure 3) promotes association of p47^phox^ with the rest of the subunits of NOX2, inducing its enzymatic activation. Mutant Huntingtin (mHtt) is found in complex with Rac1 and PI3K. Expression of mHtt is associated with hyperactive Rac1, which could further contribute to enzymatic activation of NOX2. Moreover, NOX2 is sequestered in lipid rafts together with mHtt, also associated with higher super oxide production, as indicated by the red arrow. The figure here depicts the few data currently available on NOX and HD, it does not illustrates the putative roles of NOX in HD which are discussed in the review.

Stimulation of N-methyl-D-aspartate (NMDA) glutamate receptors induces oxidant production both physiologically and in relation to excitotoxic cell death (Bindokas et al., 1996; Massaad and Klann, 2011). At first it was surmised that oxidants derived mostly from mitochondria (see discussions in Massaad and Klann (2011), Oswald et al. (2018), Wang and Swanson (2020)), but later it was established that NOX2 is activated subsequent to NMDAR ligation and constitutes the oxidant response (Brennan et al., 2009; Girouard et al., 2009; Brennan-Minnella et al., 2013; Figure 2).

**FIGURE 3:**
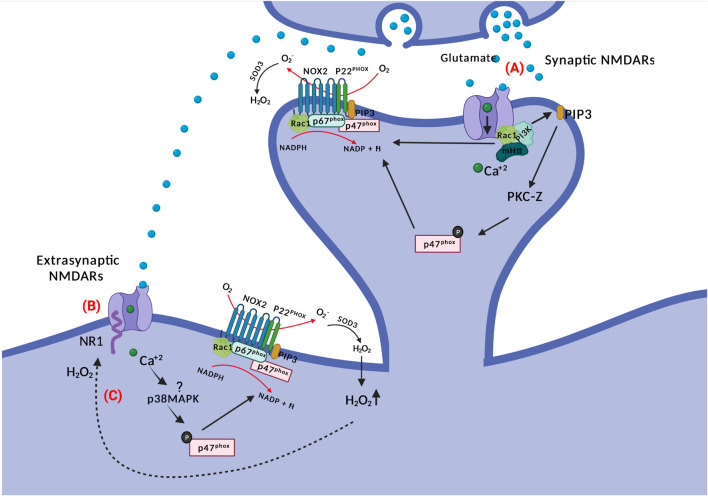
NOX and NMDAR signaling. **(A)** Glutamate binding to NMDARs activates PI3K non-ionotropically (through conformational changes in the receptor) and subsequent PIP3 production. PIP3 activates PKC-zeta, which phosphorylates p47^phox^. The latter translocates to the membrane for NOX2 complex assembly. There is also a requirement for Ca^2+^ for NMDAR-mediated NOX2 activation; potentially through Rac1. **(B)** Glutamate spillover can activate extra-synaptic NMDARs, where activated p38MAPK may phosphorylate p47^phox^ inducing ROS production by NOX2. Extrasynaptic NMDARs activation is associated with neuronal death in HD. **(C)** NR1 subunits of NMDARs are redox targets of hydrogen peroxide, which alters their function and conductivity.

Protein kinase C (PKC) is an important kinase family that phosphorylates p47^phox^ (Fontayne et al., 2002), which can be activated downstream of NMDAR activation and calcium influx. Early observations, just after the discovery of the expanded NOX family, demonstrated the necessity of both PKC and oxidant production for synaptic potentiation (Knapp and Klann, 2002), and since then, further evidence for the involvement of different PKC isoforms in NOX2 activation following NMDAR stimulation and synaptic plasticity in neurons has been provided (Brennan et al., 2009; Brennan-Minnella et al., 2013; Yi et al., 2018; Wang and Swanson, 2020).

In an *in vitro* study of mouse cortical neuronal cultures, NMDAR activation-related calcium influx activated PKC-zeta (indirectly, as this novel type PKC does not require calcium for activity) which subsequently phosphorylated p47^phox^ and caused its translocation to the cell membrane for NOX2 activation. When using neuronal cultures from transgenic p47^phox–/–^ mice, as well as when using peptide inhibitors for PKC-zeta, oxidant production and excitotoxic cell death was blocked (Brennan et al., 2009).

The evidence for activation of PKC-zeta downstream of NR2B-containing NMDARs is particularly strong (Wang and Swanson, 2020). NR2B-dependent calcium influx through NMDAR leads to activation of phosphoinositide 3-kinase (PI3K) and production of PIP3, the latter activating the atypical PKC-zeta, which then phosphorylates p47^phox^ (Brennan-Minnella et al., 2013). Excitotoxic neuron death and superoxide formation could be prevented by the PI3K inhibitor wortmannin (and p47^phox^ knockout). The involvement of PKC-zeta has been further refined, as it has been demonstrated that specifically phosphorylation at position 316 of p47^phox^ is required for induction of LTD (Yi et al., 2018). Incidentally, generation of phosphoinositide species in the vesicular or plasma membrane by PI3K is also a direct recruitment factor for p40^phox^ and p47^phox^, respectively, via their PX domains (Zhan et al., 2002; Ago et al., 2003; Ellson et al., 2006).

In phagocytes, activation of p47phox seems dominant in relation to Rac1 activation for NOX2 assembly and enzyme function (Roepstorff et al., 2008), however in artificial systems (Price et al., 2002), and potentially other cell types, Rac1 activation is sufficient to drive NOX2 activation. Tousley and colleagues have contributed with another study possibly linking NOXs and HD, through Rac1 (Tousley et al., 2019b). Rac1 activity is required for actin remodeling, a process that is required for the change in morphology and structure of dendritic spines. Moreover, actin remodeling is also necessary for the formation of new axonal branches and end-feet, therefore giving it a role in synaptic plasticity and connectivity (Spence and Soderling, 2015). Interestingly, specifically the active form of Rac1 (GTP bound Rac1) is necessary for NOX1, NOX2 and NOX3 enzymatic activation. Huntingtin is found in complex with GTP-Rac1 and p87α (component of the kinase PI3K) together with alpha-actinin1 and other proteins (Tousley et al., 2019b). This interaction is increased when mutant huntingtin (mHtt) is expressed and is accompanied by Rac1 hyperactivity. In the HD scenario, higher activity of GTP-Rac1 could be translated to an increased ROS production by Rac1-dependent NOXs (see Figure 2). Increased ROS production by NOXs could further contribute to impaired neuronal arborization dynamics, which in part is coordinated by NOX derived oxidants. The role of NOX derived oxidants in neuronal arborization is explained with more details in one of the sections of this review: “Role of NOXs in neurite growth, specification, and connectivity during development and after nerve injury”. Remarkably, hyperactivity of Rac1 is associated with dysfunctional neuritogenesis of cortical projection neurons (Zamboni et al., 2018), a neuronal subtype highly affected in HD.

Evidence has been forwarded that the activation of PI3K by NMDAR activation is non-ionotropic, meaning that although Ca^2+^-conductance is required for NOX2 activation (Wang and Swanson, 2020), PI3K is activated directly by ligand-induced conformational changes in the cytosolic aspect of NMDAR subunits alone (Minnella et al., 2018). This would provide for an exceptionally rapid activation of PKC and NOX2 assembly for oxidant production upon NMDAR ligation by glutamate, in particular if the NOX2 complex is in a primed state and active Rac1 is available.

In an excitotoxic setting, the NMDAR-stimulated oxidant release from neurons is sufficiently robust that it can induce oxidative stress even in neighboring neurons *in vitro* (Reyes et al., 2012; Brennan-Minnella et al., 2013) and cause their bystander cell death. This may also occur *in vivo*. Thus, social isolation dramatically upregulates NOX2 in pyramidal neurons of the prefrontal cortex. Nevertheless, it is the small, parvalbumin-positive GABAergic inhibitory interneurons, without NOX2 immunoreactivity, dispersed between the pyramidal neurons that are eventually progressively lost in this psychosocial stress model (Schiavone et al., 2012). When active NOX2 is surface-localized, superoxide, rather than hydrogen peroxide, for an intracellular superoxide source, will be released directly into the surroundings, where it reacts promptly and diffusion-limited with available NO to form the highly toxic peroxynitrite (NO_3_^–^), which is the main driver of oxidative damage and cell death (Wang and Swanson, 2020). A fraction of ROS is released to the surroundings, and in astrocyte-neuron co-cultures, with neurons coming from E14 mice cortices, it has been shown that NMDAR activation causes oxidative stress in neighboring neurons and glia cells (Reyes et al., 2012). Notably, this does not happen in cultures established from p47^phox–/–^ neurons, indicating the crucial role of NOX2 in the matter (Reyes et al., 2012).

Whether NMDA promotes NOX2 activation also extrasynaptically is not settled. A single study has linked NR2B (a subunit of the NMDAR receptors which is enriched in extrasynaptic sites and associated with cell death (Hardingham, 2009; Martel et al., 2009)) with superoxide production. Treatment of neuronal cultures with N2RB antagonists resulted in blockage of NMDAR-induced NOX2 superoxide production and excitotoxic cell death (Brennan-Minnella et al., 2013), indicating a crosslink between NOX and excitotoxic neuronal cell death. But interestingly, the authors showed that after blocking specifically synaptic NMDARs with MK801, activation of the remaining (extrasynaptic) NMDARs with bicuculline and 4-aminopyridine still provoked an increase in dihydroethidium (DHE) fluorescence, suggesting that extrasynaptic NMDARs can also activate oxidant generation. Moreover, adding NADPH into the bath triggered a rise in DHE fluorescence which was higher than without synaptic receptors blockage (Brennan et al., 2009), implicating NOX activity rather than mitochondria as the oxidant source. Extrasynaptic NMDAR signaling upregulates the activity of p38MAPK, and this stress kinase is known to phosphorylate and partially activate (prime) p47^phox^ and NOX2 (Brown et al., 2004; Dang et al., 2006).

## NMDAR Excitotoxicity in HD

Excitotoxic cell death, caused by overactivation of NMDA receptors, has been considered in the pathogenesis of HD, and specifically extrasynaptic NMDAR are associated with striatal neuronal loss in HD (Heng et al., 2009). Synaptic versus extrasynaptic NMDAR signaling, or hypofunction of NMDAR, already causes a reduced balance of transcription of antioxidant genes (Hardingham and Bading, 2010; Hardingham and Do, 2016). Intriguingly, the signaling processes that emanate from synaptic versus extrasynaptic NMDARs are radically different. Thus, glutamate stimulation of synaptic NMDAR initiates pro-survival pathways including the PI3K-Akt pathway and ERK signaling, suppresses death gene transcription, and stimulates transcription of antioxidant genes and CREB-dependent transcription (Hardingham and Bading, 2010). In contrast, extrasynaptic NMDAR stimulation leads to activation of p38MAPK and FOXO1 transcription factor activation, which in effect oppose the signaling pathways and transcription imposed by synaptic NMDAR signaling, including a lessened transcription of antioxidant genes. The end result is a disruption of prosurvival pathways and an altered redox balance (Hardingham and Bading, 2010; Szlachcic et al., 2015; Hardingham and Do, 2016). The situation is exacerbated by the inhibition of peroxisome proliferator-activated receptor gamma coactivator 1-alpha (PGC-1α) function by mutant huntingtin. Normally, PGC-1α directs the transcription of several antioxidant genes (St-Pierre et al., 2006), and regulates the flow of electrons through the respiratory chain in mitochondria, but through a direct interaction mutant huntingtin inhibits PGC-1α-assisted transcription (Cui et al., 2006; Weydt et al., 2006). Thus, in the HD brain the stage is set for any subsequent overproduction or unwarranted production of oxidants in time and space – e.g., from NOX activation - to do damage.

## NOX and Oxidants in the Pre-Synaptic Compartment

NOX-derived oxidants could play a role pre-synaptically by producing oxidants in close proximity to the protein machinery responsible for neurotransmission. Alternatively, oxidants produced in the post-synapse may trans-synaptically modulate pre-synaptic mechanisms such as glutamate release. Such a role is already documented for NO (Stanton et al., 2003) and is a form of synaptic plasticity. The SNARE complex is necessary for docking and fusion of the synaptic vesicles into the pre-synaptic membrane in active synapses. Interestingly, it has been shown that SNAP25, one of the components of the core SNARE complex, is specifically sensitive to oxidation by hydrogen peroxide, and that pre-exposure to 100 μM levels of hydrogen peroxide is sufficient to prevent SNARE complex assembly. Oxidants could be supplied by NOX2 in the pre-synapse itself or extrinsically by either activated microglia or post-synaptic NOX2 activity. In the latter case, oxidants would modulate pre-synaptic function across the synaptic cleft similar to other short-lived metabolites such as NO or endocannabinoids (Gerdeman et al., 2002; Stanton et al., 2003). Furthermore, protein levels of specifically SNAP25 in the pre-synaptic plasma membrane are reduced in neurons that are subjected to oxidative stress and SNAP25 knockout experiments in non-diseased cortical projection neurons (CPNs) has been shown to induce neurodegeneration (Hoerder-Suabedissen et al., 2019). Deficits in SNAP25 expression and function has been reported in HD (Smith et al., 2007). Therefore, deficits in the SNAREs machinery function of HD synapses, as a result of exposure to NOX-derived ROS, could be a potential contributor to the synaptopathology of the disease.

## NOX Involvement in Long-Term Depression/Potentiation and Synaptic Plasticity

**FIGURE 4:**
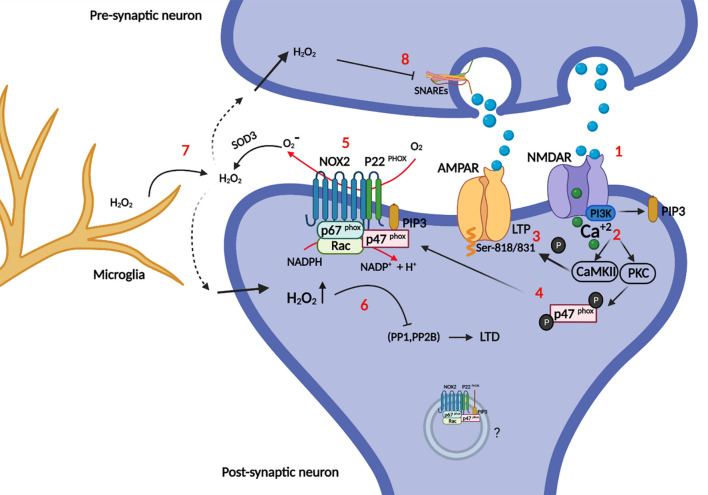
Role of NOX in synapse plasticity. Briefly, synaptic plasticity is governed mainly by LTP: Synapse enhancement through recruitment of AMPA receptors into the post-synaptic membrane and LTD: synapse weakening (AMPA receptors endocytosis). **(1)** Glutamate binding to NMDARs opens the calcium channel, and activates PI3K directly. **(2)** Calcium activates CaMKII and PKC (the latter also requires PIP3 as explained in Figure 2), which can both **(3)** phosphorylate AMPARs at specific serines, increasing their conductance and recruitment into the post-synaptic membrane (LTP). Moreover **(4)** p47^phox^ is phosphorylated by PKC, which recruits it for complex assembly and activation of NOX2. **(5)** Enzymatically active NOX2 produces superoxide in the extracellular space, which is cycled back into the cytosol as hydrogen peroxide after dismutation by SOD3. **(6)** Intracellular hydrogen peroxide potentially redox-regulates phosphatases PP1, and PP2B by oxidation. These phosphatases dephosphorylate AMPARs, promoting their endocytosis and removal from the post-synaptic membrane (LTD). The intra-synaptic pool (depicted inside a vesicle) of NOX2 may be activated differentially from the perisynaptic NOX2 in the plasma membrane and could represent a layer of specificity to redox signaling. **(7)** NOX2 derived oxidants from microglia may impair instillation of LTP or conversely promote LTD. **(8)** Hydrogen peroxide can also enter the presynaptic neuron and impair the SNAREs machinery, which is required for synaptic vesicle release.

Synaptic plasticity is in part orchestrated by long-term potentiation (LTP) and long-term depression (LTD) (Figure 4). LTP is a persistent rise in synaptic strength following high-frequency stimulation of a synapse. On the other hand, LTD is an activity-dependent decrease in the strength of synaptic connectivity. Both processes play a crucial role in the formation of specific types of memories and learning (Massaad and Klann, 2011). For example, recognition memory (the capacity to recognize formerly encountered events, objects or individuals) is processed through LTD by neural circuits in the perirhinal cortex, specifically, through an activity-dependent decrease in the efficacy of neurotransmission at glutamatergic synapses. Even though LTP and LTD effect on synaptic excitability are opposite, they can both happen at the same synapse in response to different patterns of activation of NMDARs combined with membrane depolarization. Typically, the final outcome is in one way or the other a modulation of the number and functionality of Na^+^-conducting α-amino-3-hydroxy-5-methyl-4-isoxazolepropionic acid (AMPA) receptors in the post-synaptic membrane, but other mechanisms exist (Collingridge et al., 2010).

In relation to NOX2, it was first shown that hippocampal plasticity and LTP was impaired in mouse models of CGD with no gp91^phox^ or p47^phox^ expression (Kishida et al., 2006). Studies have since then shown that oxidants and NOX2 are essential for LTP induction (see Massaad and Klann (2011) for a good historical review).

LTP can be blocked by the use of different oxidant scavengers (Lee et al., 2010), including mouse models with overexpression of extracellular SOD3 (Thiels et al., 2000), and conversely, LTP can be induced by application of xanthine/xanthine oxidase (a superoxide source) to the extracellular medium of hippocampal brain slices (Knapp and Klann, 2002). However, the latter is clearly context-dependent, as NOX2-derived oxidants from inflammatory microglia can in fact inhibit LTP induction (Wang Q. et al., 2004; Di Filippo et al., 2016). Presumably the outcome is modulated by additional reciprocal interactions between microglia and neurons (Hanisch and Kettenmann, 2007; Kettenmann et al., 2013), and it should also be noted that the effect of hydrogen peroxide concentration on LTP induction is bimodal (Kamsler and Segal, 2003).

Most studies have found that NOX2 expression in the spinal cord lies with either microglia or macrophages, where its activity plays a role in altered pain perception (Kallenborn-Gerhardt et al., 2013, 2014; Lim et al., 2013). Others have found that DRG expression of NOX4 is required for maintenance of the late phase of neuropathic pain after peripheral neurons injury, and conditional deletion of NOX4 in adult mice reduced pain-related behavior (Kallenborn-Gerhardt et al., 2012). However, one recent study proposes that LTP induction by high frequency train stimulation of the sciatic nerve in the dorsal spinal cord depends on NOX2 expression in neurons, and further describes upregulation of gp91^phox^ (Xu et al., 2020). LTP induction, but not maintenance, could be inhibited by gp91ds-tat, and further, gp91^phox^ knock-down inhibited allodynia in this mouse model of pain perception.

Post-synaptic NOX2 activity is also required for NMDAR-dependent LTD (Yi et al., 2018). LTD induced by low frequency stimulation on acute neuronal slices was dependent on post-synaptic expression of NOX2 as verified by shRNA knockdown of gp91^phox^ and p47^phox^. They also demonstrated that p47^phox^ phosphorylation at serine 316 by PKC-zeta is specifically required for LTD induction.

In some cases, oxidants do not have to be produced by neurons themselves to affect synapse function. In a pathological setting, Aβ provokes superoxide production by microglia through NOX2, and this accounts for the Aβ-induced inhibition of LTP in brain slices from mice (Wang Q. et al., 2004), and similarly, in the remitting phase of an experimental multiple sclerosis model, hippocampal LTP is impaired because of microglial NOX2 activity (Di Filippo et al., 2016). It has later been shown in an experimental LTD paradigm of combined hypoxia/LPS-stimulation in mouse models, that microglia through LPS activation of complement receptor 3 (CR3) within minutes activates NOX2 to release superoxide, which initiates a signaling cascade in the post-synapse of hippocampal neurons to down-regulate AMPA glutamate receptors, a known mechanism of LTD and synapse weakening (Zhang et al., 2014).

The opposing actions of oxidants on plasticity, dependent on context and oxidant concentration, and similarly, the participation of NOX2-derived oxidants in both LTP and LTD induction, can seem contradictory and puzzling, and clearly calls for more research into the area. A part of a reconciliatory explanation may reside in the precise subcellular localization of the oxidant source, and the differing membrane permeabilities of superoxide versus hydrogen peroxide.

In the redox signaling business proximity of oxidant producer and redox target is essential for redox modification, because the penetration range of hydrogen peroxide in cytosol is on the border of only 1–2 μm before extinction by the cellular antioxidant machinery (Winterbourn, 2008; Jones and Sies, 2015). It follows, that if two redox signaling circuits, with different outcomes, are to arise from the same oxidant source either different redox targets must be recruited dynamically to the immediate vicinity of a fixed oxidant source, or physically separated pools of oxidant producer affects different redox targets. It is therefore intriguing, that NOX2 in post-synapses is also located to endosomal elements and small unidentified vesicles and membranes (Coleman et al., 2013) in addition to the peri-synaptic surface membrane (Wang G. et al., 2004; Glass et al., 2006). Further, NOX2 activation can be directed spatially, and the proximal assembly of the holoenzyme adapted to the stimuli for activation. For example, p40^phox^ is specifically required for IgG-FcgR induced activation of NOX2 in phagocytes, where it assists the function of p47^phox^ as organizer of NOX2 assembly (Anderson et al., 2011; Ueyama et al., 2011). In addition, because of differing affinities for phosphoinositide species, p40^phox^ is recruited mainly to intracellular vesicles, whereas p47^phox^ associates with the plasma membrane (Zhan et al., 2002). In phagocytes, there is good evidence for the differential activation of cell surface resident versus intracellular pools of NOX2 (Serrander et al., 1999; Li et al., 2010), and potentially similar mechanisms could be at work in the post-synapse.

However, the penetration range of hydrogen peroxide exceeds with a good measure the size of most synapses, which speaks against a resolution of LTD/LTP-associated differential redox signaling based on physical distance alone. Another, independent or reinforcing, speculative mechanism therefore lies in the different membrane permeabilities of superoxide (charged and impermeable) and hydrogen peroxide (fairly permeable). It is curious that extracellular SOD3 abolishes LTP induction (Thiels et al., 2000), even though the enzyme converts superoxide into hydrogen peroxide, the generally accepted relayer of redox signals (Winterbourn, 2013; Sies and Jones, 2020). Could it be that specificity for one form of plasticity over the other could in part be achieved by cell surface-resident NOX2 and superoxide-mediated oxidation of cell surface proteins on the extracellular aspect? Being membrane-impenetrable, superoxide action would be restricted to redox targets on the surface in the immediate vicinity of NOX2, further reinforced by the quick dismutation to hydrogen peroxide. Only a few examples of superoxide in redox signaling are known however (Sies and Jones, 2020), and the proposed mechanism breaks with the current dogmatic view of cell surface receptor-induced NOX activation, which relies on back-diffusion of hydrogen peroxide through the membrane directly or through aquaporins to modulate intracellular redox targets. It is unknown whether synapses express aquaporins (which allows hydrogen peroxide to move along its concentration gradient), and to what extent vesicular NOX2 in synapses contributes to oxidant production, but endosomal NOX2-mediated oxidant production has been proposed in other cell types (Oakley et al., 2009; Lamb et al., 2012), and NOX2 storage vesicles also have oxidant production capability (Moreland et al., 2007; Ejlerskov et al., 2012).

The redox targets of NOX2 in synapses are entirely unknown. However, a major function of NOX2 is to regulate, by oxidative inactivation, the activity of both tyrosine and serine/threonine phosphatases (Sies, 2014). LTD and LTP are governed by a balance of kinases like PKC and Ca^2+^-calmodulin-dependent protein kinase II (CaMKII) (promotes LTP), and opposing phosphatases calcineurin (PP2B), PP1 and PP2A (promotes LTD) (Collingridge et al., 2010). Phosphorylation of AMPA receptors increases conductance and their synaptic incorporation during LTP, while dephosphorylation promotes their endocytosis, and LTD and synapse weakening. Interestingly, calcineurin, PP1A, and PP2A are all inactivated by hydrogen peroxide (Rhee et al., 2000). and NOX2 derived oxidants could activate PKC (in a feedback loop) (Knapp and Klann, 2002), and similarly, Akt, which also phosphorylates and activates p47^phox^ (Hoyal et al., 2003), is in part positively regulated by redox modification (Luo et al., 2012) (Su et al., 2019). Finally, NMDARs are targets for redox modification themselves. Redox modifications of cysteine residues in the cytosolic tails of particularly NR1B modulate NMDAR function (Lipton et al., 2002), and there is evidence that NOX2 contributes to this process (Di Maio et al., 2011). Interestingly, evidence for NOX2-derived oxidant alteration of the stoichiometric composition of the NMDAR (upregulation of NR2B) has been presented following cholinergic stimulation of m1R metabotropic receptors (Di Maio et al., 2011). In addition, metabotropic receptors can be redox modified (Campanucci et al., 2008; Coddou et al., 2009).

Outside of synapses it has been shown that NOX2 activity is required for axonal development by causing Ca^2+^ release from endoplasmatic reticulum through Ryanodine receptors (Wilson et al., 2016). However, it was not investigated whether the ryanodine receptor was a direct target of redox modification, as observed in other cell types where evidence of NOX4 participation is particularly strong (Sun et al., 2011). Notably, store operated calcium entry is affected in HD (Czeredys, 2020) likely downstream of an interaction between mHtt and the IP3R1 receptor in the rER, which sensitizes the IP3R calcium channel, such that calcium in rER is depleted and subsequently activates the store operated entry of calcium (Wu et al., 2016).

## NOX2, LTD/LTP and HD Pathology

The memory deficits that are characteristic of HD, and demonstrated in patients as well as mouse models, include failure in recognition memory (Giralt et al., 2011), and cognitive dysfunction (Milnerwood et al., 2006) which are manifest early in the disease (Montoya et al., 2006).

Electrophysiological measurements in brain slices from several different genetic mouse models have consistently shown LTP dysfunction in HD, not only the most commonly characterized hippocampal LTP (Hodgson et al., 1999; Usdin et al., 1999; Murphy et al., 2000; Quirion and Parsons, 2019) but also in the cortico-striatal synapse (Kung et al., 2007; Dallérac et al., 2011; Plotkin et al., 2014; Sepers et al., 2018). The effect of mutant huntingtin on the ability to evoke stimulation-induced LTD, also studied in various HD mouse models, is less clear. LTD evocation has been shown to be diminished in the perirhinal cortex in the R6/1 mouse model (Cummings et al., 2007) and in cortico-striatal synapses (Ghiglieri et al., 2019; Kim et al., 2020), but reports of similar changes in hippocampus has not been consistent (Murphy et al., 2000; Milnerwood and Raymond, 2007; Ghilan et al., 2014).

Different mechanisms have been proposed to convey these effects, including changes in dopamine receptor function and endocannabinoid receptor activity (Sepers et al., 2018; Ghiglieri et al., 2019). Dopamine D1 receptor activity has been shown to regulate the expression and activity of NMDAR, which as described above is essential for LTP and LTD; as D1 receptor hypersensitivity has been shown in HD this presents a potential mechanism for the involvement of mutant huntingtin in synaptic plasticity (Ghiglieri et al., 2019). Another potential mechanism involves the “alternative” brain-derived neurotrophic factor (BDNF) receptor p75^NRT^: BDNF exerts neurotrophic actions and stimulates LTP and LTD through activation of the TrkB receptors, however, BDNF binding to p75^NRT^ has been proposed to have antagonistic effects on synaptic plasticity (Brito et al., 2014). Interestingly, increased protein levels of p75^NRT^ has been shown in HD patients and mouse models (Brito et al., 2014), and in cortico-striatal synapses HD-associated abnormalities in LTP were restored by inhibiting p75^NRT^ (Plotkin et al., 2014). p75^NRT^ activation acts through PTEN, which, as described previous in this review, may be inhibited by NOX-derived ROS (Le Belle et al., 2011).

## Microglia and NOX2 in HD

Glial cells might be contributors to the pathology of HD (Wilton and Stevens, 2020), and when it concerns NADPH oxidases, by far, the most literature on NOX2 in the brain involves microglia, the resident CNS phagocytes, which additionally express NOX1 (Cheret et al., 2008). As phagocytes, they are equipped for high level oxidant production following pathogen encounter, and the very same pattern of recognition receptors come into play in the recognition of extracellular amyloids (Fellner et al., 2012; Qiao et al., 2015; Wang et al., 2015).

Relative to the common neurodegenerative diseases like Alzheimer’s (AD) and Parkinson’s disease (PD), the mass of literature on microglia in HD is limited. Search terms ‘AD/PD/HD’ AND ‘microglia’ retrieves 5755, 2600, and 257 records from PubMed, respectively. The information on NOX2 activity in microglia of animal models of HD is virtually non-existent and has only been addressed peripherally *in vitro*.

This despite the fact that PET scanning of HD patients with peripheral benzodiazepine receptor ligands (11C-(R)-PK11195), as measure of microglia activation, indicates that microglia are activated well before clinical disease onset (Pavese et al., 2006; Tai et al., 2007). Post mortem HD brains show typical signs of neuroinflammation including cytokine production and complement deposition (Singhrao et al., 1999) as well as markers of oxidative stress (see references in Bono-Yagüe et al. (2020)).

Transcriptional profiling of AD brain shows a clear inflammatory signature (Block et al., 2007) (Malik et al., 2015), and a significant correlation between NOX activity and cognitive decline exists (Ansari and Scheff, 2011). In PD models, dopaminergic neuron death can be instilled by the mere stereotactic infusion of LPS into the substantia nigra to activate microglia and NOX2 specifically (Qin et al., 2004).

The situation is somewhat different in animal models of HD, where microglia activation and neuroinflammation seems more subdued. While microglia in early disease in the R6/2 HD model decrease ramification and assume more activated shapes, indicating that microglia are detecting (Kraft et al., 2012) and responding to a deviation from homeostasis, possibly derangement of synaptic function (Milnerwood and Raymond, 2007), a neuroinflammatory profile with full blown microglia activation and proinflammatory cytokine production is observed only late in the disease (see Wilton and Stevens (2020) for an overview). At this point microglia activation is not necessarily disease-specific but represents a prototypical response to neuronal death processes, particularly so in the R6/2 mouse model where the course of disease is very aggressive (mice die at 16 weeks of age). Therefore, the recent demonstration that shRNA knock-down of Galectin 3 in R6/2 mice dampens microglia activation, neuroinflammatory profile, and ameliorates disease (increasing survival) is surprising (Siew et al., 2019). It will be interesting to see whether microglia activation also plays out as a driver of pathology in other HD animal models with a more protracted disease course than R6/2 mice (Plotkin and Surmeier, 2015).

In contrast to idiopathic brain disease, where microglia are typically initially activated down-stream of neuronal dysfunction or extracellular amyloid aggregates, all glia cells in HD endogenously express mutant huntingtin, which affects their activation and function from within (Wilton and Stevens, 2020). In the R6/2 model, microglia display pro-inflammatory transcriptional activation by increasing the expression and transcriptional activities of the myeloid lineage-determining factors PU.1 and C/EBPs (Crotti et al., 2014). Of note, increased transcription of PU.1 and C/EBP target genes was microglia specific in the R6/2 model, which contrasts with the monocytic priming by mutant huntingtin expression observed early in human patients (Bjorkqvist et al., 2008). It has also been noted that microglia, and myeloid cells in general, in experimental HD models or patients show a reduced migratory response versus chemotactic stimuli *in vitro* or laser-induced injury *in vivo* (Kwan et al., 2012). However, selective depletion of mutant huntingtin in myeloid cells, including microglia, has no bearing on disease development in the BACHD HD mouse model (Petkau et al., 2019).

Whereas both amyloid-β (Bianca et al., 1999; Zhang et al., 2011) and α-synuclein (Fellner et al., 2012; Qiao et al., 2015; Wang et al., 2015) aggregates induce NOX2-dependent superoxide production in microglia through specific scavenger receptors, no such data are available for mutant huntingtin aggregates. In fact, no internalization or signaling cell surface receptors are known for mutant huntingtin in microglia (or in any other cell type for that matter). To date therefore, no publications have specifically addressed microglial NOX-mediated oxidant production in an *in vitro* or *in vivo* setting of HD models, discounting studies that, too optimistically, rely only on apocynin or other general anti-oxidant inhibition as measure of NOX involvement (Maldonado et al., 2010; O’Regan et al., 2021).

## Conclusion

While the role of microglia and NOX2-mediated oxidant production in the progression of late stage neurodegenerative disease is undisputed, the involvement of neuronal NOX isoforms in physiology and brain pathology are just in the process of being unraveled. From the early recognition of a requirement for superoxide for LTD/LTP instigation, several groups have now contributed to the delineation of the pathways that lead to NOX2 activation following NMDAR engagement in synaptic parts of neurons. However, the redox circuits downstream of NOX2 oxidant production, and in particular how both LTD and LTP are redox controlled by NOX2, awaits further elucidation, and presumably identification of protein redox targets. It will also be interesting to see whether the deleterious effects of extrasynaptic NMDAR signaling are mediated in part by NOX2 activity and by which pathway; if different from synaptic NOX2 activation they could perhaps be uncoupled pharmacologically.

Any specific effects of neuronal NOX activity in HD would depend in the (1) stage of the disease, (2) specific cell type and NOX isoform involved, and (3) subcellular localization of the enzyme – the latter given the complex neuronal morphology and extensive length of the axons, allowing for several redox signaling microcircuits to occur within the same cell. And last but not least, (4) NOX-derived ROS from other cell types, in this particular case microglia, may also play a role in HD disease progression, which has not been fully explored yet.

We have presented evidence that NOX function could potentially participate from the very start of developmental defects seen in HD, including altered neurogenesis, neurite growth, and axonal pathfinding, while also having a possible role in mature neurons where NOX2 regulates NMDAR signaling and synaptic plasticity, known to be affected in presymptomatic HD. It will be essential to follow up on the present isolated findings of mHtt modulation of PI3K and Rac1 signaling, as it will be important to establish whether mHtt interacts with NOX2 in lipid raft compartments of the membrane to interfere with crucial synaptic processes that mediate synaptic plasticity and maintenance. To the extent that the “dying back” pattern of degeneration in which loss of synaptic connectivity and axonal degeneration precedes and provokes neuronal death over time (Han et al., 2010), a lot could potentially be gained by remedying initial synaptic dysfunction in the striatum, which would also presumably ameliorate early motor symptoms.

We hope that it can be conjectured from the present review that the relative ‘under-representation’ of literature on NOX in HD compared to other NDDs cannot simply be explained by the different prevalence of these diseases; there is certainly room for future research into the roles of NOX and oxidants in HD.

## Author Contributions

All authors listed have made a substantial, direct and intellectual contribution to the work, and approved it for publication.

## Conflict of Interest

The authors declare that the research was conducted in the absence of any commercial or financial relationships that could be construed as a potential conflict of interest.

## Publisher’s Note

All claims expressed in this article are solely those of the authors and do not necessarily represent those of their affiliated organizations, or those of the publisher, the editors and the reviewers. Any product that may be evaluated in this article, or claim that may be made by its manufacturer, is not guaranteed or endorsed by the publisher.
